# Stress response of fire salamander larvae differs between habitat types

**DOI:** 10.1098/rsos.231304

**Published:** 2024-04-03

**Authors:** Laura Schulte, Pia Oswald, Max Mühlenhaupt, Edith Ossendorf, Sabine Kruse, Sylvia Kaiser, Barbara A. Caspers

**Affiliations:** ^1^ Behavioural Ecology Department, Bielefeld University, Bielefeld 33615, Germany; ^2^ Institute for Neuro- and Behavioural Biology, University of Münster, Münster 48149, Germany; ^3^ Joint Institute for Individualisation in a Changing Environment (JICE), University of Münster and Bielefeld University, Bielefeld, Germany

**Keywords:** water-borne corticosterone release, niche conformance, *Salamandra salamandra*, reciprocal transplant experiment, corticosterone phenotype, stress physiology

## Abstract

The larvae of the European fire salamander (*Salamandra salamandra*) can inhabit two different habitats: streams and ponds. Streams are characterized by lower predation risks and higher food availability. Thus, ponds are considered a less suitable habitat. To investigate the differential impacts of these two habitats on larval physiology, we measured the stress response of larvae. After successfully validating the measure of water-borne corticosterone release rates in fire salamander larvae, we measured the baseline and stress-induced corticosterone of 64 larvae from ponds and streams in the field. We found that larvae in ponds have a higher baseline and stress-induced corticosterone levels. Additionally, we performed a reciprocal transplant experiment (RTE) and tested whether larvae can adapt their stress responses to changing habitats. After two weeks, we did not find an increase in corticosterone levels when comparing stress-induced corticosterone values with baseline corticosterone values in larvae transferred into ponds, irrespective of their habitat of origin. However, larvae transferred into streams still exhibited an increase in the stress-induced corticosterone response in comparison with the baseline values. These results show that non-invasive hormone measurements can provide information on the habitat quality and potential adaptation and thus emphasize the potential for its use in conservation efforts.

## 1. Introduction

Individuals of the same species often reside in different habitats, which can come with different selection pressures. For instance, the common raven (*Corvus corax*) inhabits different habitat types and adapts its diet to food resources available in each type of habitat [1]. Other species show adaptive morphological changes in response to different habitats such as the Mexican tetra (*Astyanax mexicanus*) [2]. This fish species is found in both surface and cave waters and has adapted to very different environments such as reduced or no eyes in the cave dwellers. Selection pressures might also shift due to environmental changes such as human-induced climate changes. For example, precipitation becomes unpredictable and unstable, causing droughts and floods. These extreme weather events will probably occur more frequently in the future and can substantially impact aquatic ecosystems in particular [3–5]. Stagnant water bodies, for example, are expected to dry out and become less abundant in the future due to droughts, while floods might lead to overflooding of streams, causing mass-drift events [3]. Therefore, conforming to a changing environment, i.e. adjusting the phenotype of an individual to a given environment [6], imposes great challenges for organisms, both now and in the future.

Adjusting to a changing environment is particularly challenging for amphibians, because most of them rely on water bodies for reproduction, and strong population declines have already been observed due to human-induced environmental changes [3,4,7,8]. Additionally, they need to adapt to the habitat type according to their current developmental stage, which is often aquatic before and terrestrial after metamorphosis [9]. Environmental changes can influence the habitat quality in different ways. Less precipitation, for example, can lead to quicker desiccation of ephemeral water bodies. However, knowledge regarding which habitat type is less stressful for a species is often missing. Environmental stressors have the potential to alter the level of glucocorticoids [10–13]. Hence, one way to evaluate stress responses in vertebrates is to measure the release of glucocorticoids. Glucocorticoid (GC) hormones, such as cortisol and corticosterone, are useful biomarkers to assess the condition of amphibians, for example, they can predict reproductive success [14]. GC hormones are responsible for homeostasis and growth levels and have immune effects, and corticosterone is the most responsive GC hormone for most amphibians [15–17]. Additionally, they are responsible for changes in morphology, physiology and behaviour and can also disrupt the amphibian life cycle [10,18–21].

Long-term stress can be assessed by measuring the baseline corticosterone release, while the stress response towards an acute stressor can be assessed by measuring stress-induced corticosterone release [17,22]. When stress hormones are chronically elevated, they can negatively impact organisms and lead to higher disease rates [23,15,20,24–26]. However, when elevations are acute, they can have positive short-term effects such as increased immune responses and survival [12,13,27]. Additionally, the resilience towards environmental stressors such as predation or climatic challenges, can be measured as stress responsiveness [28].

In general, amphibian larvae show great phenotypic plasticity towards abiotic and biotic factors in their habitat. This can be due to environmental changes such as desiccation [29] or anthropogenic influence [18]. Organisms that show high levels of plasticity will possibly be able to respond faster to a changing and unstable habitat [30]. *Bufo bufo* tadpoles, for example, showed a different endocrine plastic response towards different environmental conditions [18]. Habitat conditions can influence the endocrine system which in turn has a major impact on the plasticity of individuals. Thus, the larval habitat and/or different larval habitats, respectively, can indirectly influence their fitness via the endocrine system [31]. Hence, investigating the individual baseline and stress-induced corticosterone release will provide insights into potential long-term environmental stressors and the ability of individuals to cope with acute stress. A non-invasive and non-lethal way to assess the stress response of an organism is water-borne hormone sampling [15,32]. In the past, corticosterone levels were often measured by euthanizing animals, especially small organisms [32,33]. With this method, the hormones can be sampled from the water as amphibians release hormones passively through their gills and urine [32]. Thus, measuring the water-borne hormone release rate is a promising alternative and particularly beneficial for small aquatic or semiterrestrial organisms [17]. Moreover, it allows for repeated sampling [17,32], providing the opportunity to study individual conditions over a longer period or under changing conditions.

The larvae of the European fire salamander (*Salamandra salamandra*) are present in very different habitats. They are deposited mainly into first-order streams [34] and also into ephemeral ponds [35,36]. Interestingly, pond-adapted females deposit their larvae more often and over a longer period of time than females depositing larvae into streams [37]. These habitats differ in several aspects. Salamander larvae in streams face little predation pressure [38], while the predation pressure in ponds is generally higher [34,36]. The analyses of potential food resources for fire salamander larvae have shown that streams offer greater energetic values of potential food than ponds [39]. Additionally, the densities of conspecifics in ponds are much higher than those in streams [36]. Laboratory studies found that the amount of food available to larvae influences risk-taking behaviour [40] and the amount of yellow coloration after metamorphosis [41], both having potential fitness consequences. Additionally, a previous reciprocal transplant experiment (RTE) showed that larvae in pond habitats have higher growth and post-metamorphosis survival rates than larvae in stream habitats [42]. In summary, it is not yet understood which larval habitat reflects less stressful conditions for the larvae: ponds or streams.

The larval stage represents the most critical time during the fire salamander life cycle, as fire salamanders rely on aquatic habitats for reproduction (except for some Iberian subspecies, see [34]). Metamorphosed salamanders have almost no natural predators and live in more stable habitats, as water bodies are only important for larval deposition and fire salamanders can survive long periods of drought in the soil or underneath deadwood [34,43,44], but see [45]. Thus, the larval stage should be the focus of conservation measures. Considering the environmental changes caused by global warming and extreme weather events, it is important to understand which larval habitat is more stressful for fire salamanders in light of conservation physiology, as both habitat types will be differently affected by extreme weather conditions.

In this study, we aimed to quantify how larvae from two habitat types (ponds and streams) differ in their stress physiology. Additionally, we performed an RTE in which we transferred larvae between these two habitat types. Thus, we assessed whether larvae could adapt to changing habitat conditions. We investigated (i) differences in the stress responses between pond and stream larvae, and we expected pond larvae to have higher baseline corticosterone release levels than stream larvae due to harsher conditions in ponds. Furthermore, we wanted to test whether larvae are plastic in their stress response and can conform to the given conditions of a specific habitat. If this is the case, we investigated (ii) that the transfer habitat influences the stress response, whereas if larvae cannot conform to the given habitat, we expected to see an effect of the original habitat on larvae after the RTE.

## 2. Methods

### 2.1. Study area

**Figure 1.**
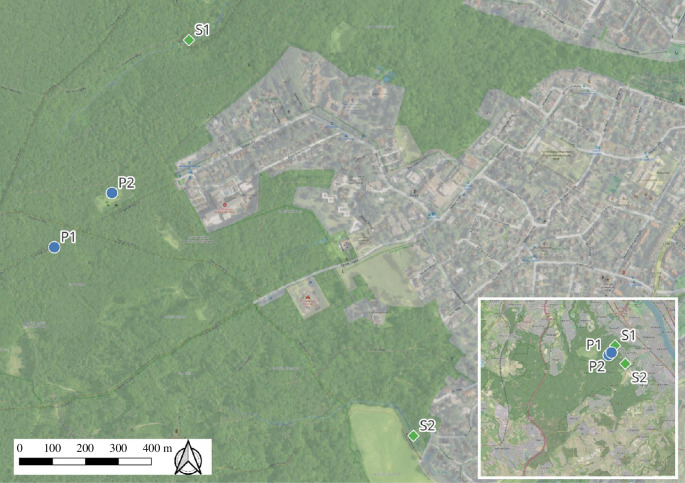
The four study locations in Kottenforst, Bonn, Germany. The two blue dots represent the two pond locations (P1 and P2) and the green squares represent the two stream locations (S1 and S2). The inlet provides a larger overview of the Kottenforst.

The study sites are located in Kottenforst, Bonn, Germany (50°39’30.58” N, 7°4’4.70” E, figure 1). The forest mostly consists of *Luzulo-Fagetum* and *Stellario-Carpinetum* communities with European hornbeam (*Carpinus betulus*), common oak (*Quercus robur*) and European beech (*Fagus sylvatica*) as the main tree species forming an ideal habitat for fire salamanders [34]. In some parts of the forest, however, Scotch pine (*Pinus sylvestris*) and European spruce (*Picea abies*) dominate the landscape. The Kottenforst holds several streams and contemporary ponds both used for larval deposition by fire salamander females [35,36,46,47]. These two different habitat types are associated with two different genotypes of the fire salamander, indicating a recent local adaptation [35].

### 2.2. Water-borne hormone sampling

**Figure 2.**
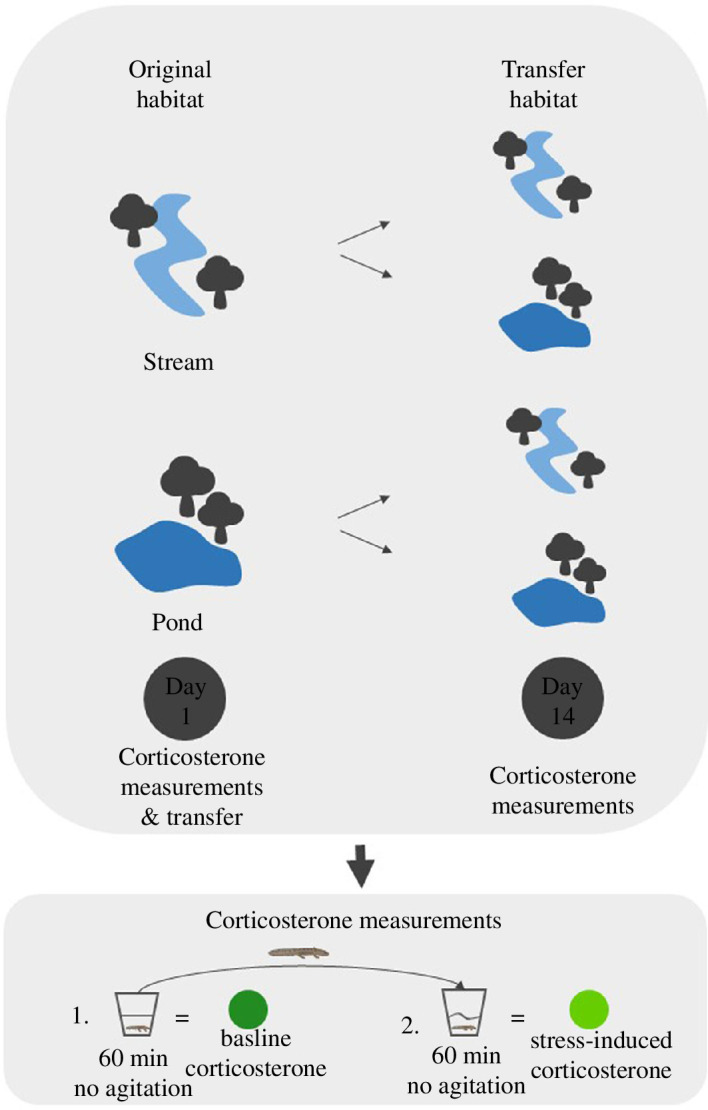
Top: on day 1 of the experiment, larvae were tested for baseline (60 min without agitation) and stress-induced (60 min with agitation) corticosterone levels immediately after capturing them from their original habitat. Afterwards, they were transferred reciprocally among four locations (two ponds and two streams, including the location of origin). On day 14 after the transfer, larvae were measured again for baseline and stress-induced corticosterone levels, this time at the transfer location. Bottom: for the corticosterone measurements, larvae were first tested for baseline corticosterone levels. We took the larvae from the water body with a dip net. We immediately placed them in a plastic container with 40 ml of tap water and maintained them for 60 min. Afterwards, we placed them in 40 ml of fresh tap water to assess stress-induced corticosterone levels. Here, we again maintained them for 60 min, but this time we agitated the containers for 1 min every 3 min.

In April 2022, we tested 64 fire salamander larvae for baseline and stress-induced corticosterone release rates using water-borne hormone sampling. We captured larvae from four locations, two ponds and two streams (*n* = 16 from each location, figure 1). None of the captured larvae showed the signs of metamorphosis (e.g. reduced gills) or were morphologically impaired (e.g. missing tails or missing limbs); consequently, all the captured larvae were used in the study. Immediately after capture, we placed the larvae individually into a 400 ml plastic container with 40 ml of tap water each (figure 2). All tap water used was taken on the same occasion and thus was identical for all sampling locations. We did not treat the tap water (e.g. dechlorinate), as the tap water in Germany is of good quality. Each larva was kept in the container for 60 min to measure the baseline corticosterone release rate per hour (altered after [25,48,49], figure 2). We newly validated the corticosterone measurements for fire salamander larvae as reported by Gabor *et al*. [32].

The containers were placed in the shade and left undisturbed for the first 60 min. After 60 min, we transferred each larva into a new plastic container with 40 ml of fresh tap water and maintained for another 60 min and agitated them manually for 1 min every 3 min to measure stress-induced corticosterone release [48]. The water from the first and second plastic containers was transferred separately into 50 ml Falcon tubes and stored in a portable car freezer (−10°C) immediately after conducting the experiment in the field (approximately 2.5 h after starting with the baseline corticosterone release). After returning from the field, we stored the samples at −20°C until further processing. We cleaned all plastic containers with 70% ethanol and rinsed them with the same tap water used for the experiment [49]. Sampling was conducted during different times of the day and we altered the time of the experiments between the two habitats to control potential circadian variations in corticosterone release levels. We also collected negative and positive hormone control samples to assess the quality of the assay (see below). Negative control samples consisted of 40 ml of tap water, which was the same tap water that we used for corticosterone measurements. They were also frozen at −20°C until the water-borne corticosterone concentrations were determined. We produced two different positive control samples by pooling the samples of 21 larvae prior to this experiment. The positive control samples were aliquoted into 40 ml vials and maintained at −20°C. We wore nitrile gloves during the entire experiment. After the corticosterone release rate measurement, we photographed the larvae from the right body side and the top, and we measured the total length in mm (size). We sterilized all our equipment with *Virkon S* (LANXESS Biosecurity) before and after sampling to avoid the potential spread of the chytrid fungus *Batrachochytrium salamandrivorans* in our study area.

### 2.3. Reciprocal transplant

To investigate whether the larvae can alter their corticosterone release rate to the changed habitat, we performed an RTE [50,51]. Briefly, within our two habitat types (ponds and streams), we used four locations (two streams and two ponds), resulting in the following treatment groups: stream-origin transferred into streams (St/St), stream-origin transferred into ponds (St/P), pond-origin transferred into ponds (P/P) and pond-origin transferred into streams (P/St) (figure 2).

To control the location and transfer effects, we transferred four larvae from each location into the same habitat type but into a different location, four from each location to each of the two locations of the other habitat type and four remained in their original habitat (same location). After performing the measurements as described above (water-borne hormone sampling), we placed the larvae into individual enclosures (8.5 × 8.5 × 17 cm, similar to [51]) and assigned them randomly to one of the treatment groups and locations. We obtained a repeated measure of water-borne corticosterone levels after two weeks followed by an agitation stressor for 1 h (figure 2). The individual enclosures had small grids that allowed food items to enter the enclosure and prevented the larvae from escaping. Additionally, they had styrofoam pieces attached to the top to enable floating and we equipped the enclosures with a stick to prevent drowning in case the larvae underwent metamorphosis. We checked the larvae every day to see if they were alive or about to undergo metamorphosis. All larvae were released after two weeks of the experiment.

### 2.4. Water-borne hormone extraction and determination of corticosterone levels

After thawing, the water-borne hormone samples were centrifuged (5 min, 2500*g*). Hormones were then extracted from the supernatant under vacuum pressure using a Chromabond SPE vacuum chamber and HR-X SPE (solid phase extraction) columns (CHROMABOND^®^ HR-X polypropylene columns, 3 ml/60 mg/45 µm; Macherey-Nagel [no. 730936 P45], Düren, Germany). The columns were activated with 3 ml of methanol (p.a.) and 3 ml of demineralized water, which are sufficient for conditioning (see recovery rate, electronic supplementary material, table S5). After activation, the columns were filled with 2 ml of demineralized water. The samples were then added to the column using a 70 ml reservoir tube connected to the columns. The vacuum was regulated in such a way that samples were adsorbed to the HR-X columns at a flow rate of less than or equal to 3 ml min^−1^. After running the samples, the columns were first washed under maximum vacuum pressure with a column volume (i.e. 3 ml) of demineralized water, then pulled as dry as possible, sealed with Parafilm and stored temporarily at −20°C. After thawing the columns, the elution of corticosterone was performed with 1 ml of methanol (p.a.) and an elution rate of less than or equal to 1 ml min^−1^. As tested before, this elution volume was sufficient to elute the bound corticosterone from the column (see recovery rate, electronic supplementary material, table S5). Methanol was evaporated directly afterwards in a vacuum concentrator and corticosterone was resuspended in 250 µl of ELISA buffer in the test tubes (Cayman Chemicals Inc. [no. 501320]). The samples were vortexed and the tubes were pulled vigorously over a stainless steel tube rack. The samples were stored at −20°C for a minimum of 16 h until ELISA was performed. After thawing, the samples were vortexed again. Corticosterone concentrations were determined in duplicate and according to the manufacturer’s instructions. Each ELISA plate was randomly loaded with samples from two larvae from each area (St/St, St/P, P/P and P/St). In addition to these 32 samples, two positive and four negative controls (two samples of tap water from the original habitat and two samples of tap water from the transfer habitat) were added per plate.

### 2.5. Assay quality control

#### 2.5.1. Cross-reactions and inter- and intra-assay variations

Two positive control samples were used to determine the inter-assay variation. One sample could be determined in duplicate on a total of six plates and the second on eight plates. The inter-assay deviation was an average of 7.7%. Furthermore, four pools of different hormone concentrations were used to determine the intra-assay variance: corticosterone concentrations of each pool were determined 12 times for one plate, resulting in an average intra-assay coefficient of 6.6%. Finally, all samples were assayed in duplicate and the determination of the sample was repeated if the CV was larger than 10%. The antibody showed the following cross-reactivities: corticosterone 100%, 11-deoxycorticosterone 15.8%, prednisolone 3.4%, 11-dehydrocorticosterone 2.9%, cortisol 2.5%, progesterone 1.4%, aldosterone 0.47%, 17α-hydroxyprogesterone 0.21%, 11-deoxycortisol 0.14%, androstenedione 0.11% and all other tested steroids less than 0.1%.

#### 2.5.2. Linearity/parallelism

To assess linearity, we ran a serial dilution of three pooled samples in duplicate (see electronic supplementary material, table S3).

#### 2.5.3. Recovery rate

Two different recovery rates were determined:

1. After extracting corticosterone from the water, the samples were resuspended in ELISA buffer (see above). Three pooled samples of different concentrations resulting from these resuspended samples were used to calculate the recovery rate of the assay. We conducted spikes by mixing equal volumes of the respective pooled sample with three different standards, which were also used for the standard curve in the ELISA. In addition, we determined the concentrations of the non-spiked, non-diluted and 1 : 2 diluted pooled control samples. All samples were assayed in duplicate. The expected recovery concentrations were based on the known amount of corticosterone concentrations in the control samples (see electronic supplementary material, table S4).
2. To assess the recovery rate of the HR-X SPE columns, we used the following procedure: four different standards, which were also used for the standard curve in the ELISA, were added to tap water and the same procedure as described above was conducted. Furthermore, we prepared water samples containing each of the four different standards and a pooled sample. The concentrations of the pooled sample were determined in the same assay. All samples were assayed in duplicate. The expected recovery concentrations were based on the known amount of the standards and pooled samples minus the concentration of the negative controls (see electronic supplementary material, table S5).

#### 2.5.4. Calculation of corticosterone concentrations

The concentrations of corticosterone in pg ml^−1^ measured in the ELISA were multiplied by 0.25 ml (the volume of ELISA buffer used to resuspend the sample) after the concentrations of negative control samples were subtracted from the sample values (a standard procedure that must be used because of matrix effects) [33]. These values were standardized by dividing them by the body size of each individual. Thus, corticosterone concentrations were given as ‘pg cm^−1^ h^−1^’.

#### 2.5.5. Statistical analyses

The corticosterone measurements were first standardized to the size as pg cm^−1^ h^−1^ [32]. We tested for normality using the Shapiro–Wilk test and homogeneity of variance using the variance test (*dplyr* R package). If normality was not found, we used the *bestNormalize* R package to find the best transformation procedure for our data to fulfil the normalization assumptions. For linear models (LMs) and linear mixed effect models (LMMs), we used the R packages *lme4* and *lmerTest* [52,53].

The first sampling point, that is, before the RTE, aimed to test for a potential impact of the habitat on baseline corticosterone release and stress-induced corticosterone release. Therefore, we performed an LMM with corticosterone release as the dependent variable and corticosterone type (baseline corticosterone or stress-induced corticosterone) and habitat (ponds and streams) and the interaction between the two as fixed factors. We controlled repeated measurements and potential location effects (e.g. sibling effect) using an individual ID and a sampling location as random factors. We included temperature in one of the models, but it was not best supported by the Akaike information criterion(AIC) and the best performance package. Also, we found no significant difference between the habitat types.

Similarly, we used another LMM to investigate if there was an impact of the habitat on the baseline and stress-induced corticosterone release rates, this time after the RTE. We included both the habitat of origin and the transfer habitat in the model and the corticosterone type as fixed factors, and the interaction between the corticosterone type and the transfer habitat, and the corticosterone type and the habitat of origin. We used the ID and transfer location as random factors to control repeated measurements and different habitat conditions in the transfer location.

To test for habitat-specific differences between baseline and stress-induced corticosterone levels, we used two LMMs, one for ponds and one for streams both after the transfer, to test for the differences in corticosterone between the corticosterone type (baseline and stress-induced corticosterone) as fixed factors, and ID and location as random factors. To investigate whether pond and stream larvae differ in their baseline or stress-induced corticosterone levels, we compared only baseline corticosterone levels between ponds and streams and only stress-induced corticosterone levels between ponds and streams after the transfer. In addition, we compared the deviation in baseline and stress-induced corticosterone levels (= 100 × (stress-induced corticosterone − baseline corticosterone)/baseline)) [18] after the transfer per treatment group (P/P, P/St, St/P and St/St), and we used the LM to test against the treatment groups. As a *post hoc* test, we used the package *emmeans* to compare the different treatment groups. Finally, we used another LM to investigate the differences in the absolute growth rate among the treatment groups (P/P, P/S, St/P and St/St) after the transfer using *emmeans*. To test if there was a difference in the size of the larvae from the two habitat types before the transfer, we performed a *t*-test. We conducted all statistical analyses with R Studio (version R 2022.02.3). We set the significance level to α = 0.05. No data were excluded, but three corticosterone measurements (1 × baseline, 1 × stress-induced corticosterone release rate from one pond larva before the transfer and 1 × stress-induced corticosterone release rate from one stream larva after being transferred into a pond) were lost due to the handling error. All graphs were produced using the package *ggpubr* [54].

#### 2.5.6. Limitations of the study

Newcomb Homan *et al*. [55] found a different stress response in adult female and male spotted salamanders (*Ambystoma maculatum*). For fire salamander larvae, it is not possible to determine sex and sex ratios in wild populations are unknown, which is why we cannot control potential sex effects. Other studies, however, have not found the sex effect on the stress response in a wild population of adult San Marcos salamanders (*Eurycea nana*) [56].

## 3. Results

### 3.1. Before the transfer

**Figure 3.**
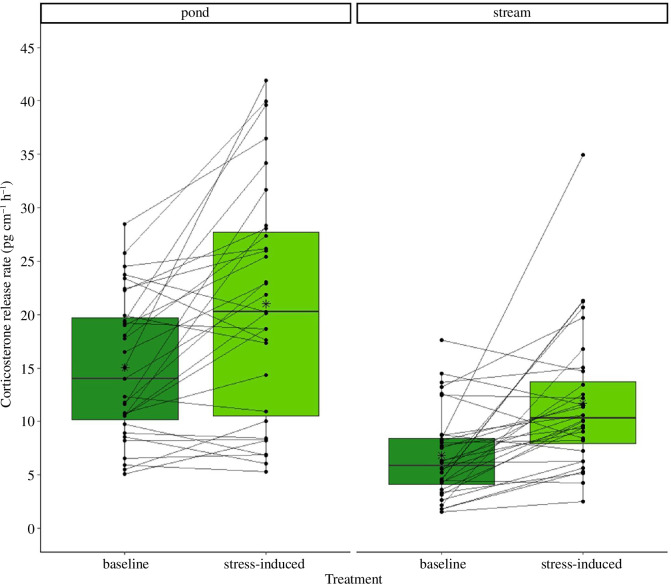
The water-borne corticosterone release rate (pg cm^−1^ h^−1^) in fire salamander larvae (*Salamandra salamandra*) before treatment. Stress-induced corticosterone release rates were significantly higher than baseline corticosterone release rates in both pond (baseline *n* = 31 and stress-induced *n* = 31) and stream (baseline *n* = 32 and stress-induced *n* = 32) larvae before the transfer. Each point represents a single corticosterone measurement and each line connects the two measurements per individual. The horizontal line represents the median and the asterisks in the box represent the mean.

We found no difference in the size of the larvae between the pond and stream habitats of origin (*t*-test and *p* = 0.738). The stress-induced corticosterone release rate was significantly higher than the baseline corticosterone rate (*p* < 0.001, table 1) independent of the habitat (figure 3). Additionally, overall corticosterone release rates (baseline and stress-induced corticosterone) were significantly lower in streams than those in ponds (*p* = 0.01). We did not find an interaction effect between the habitat type (ponds or streams) and the corticosterone type (baseline or stress-induced corticosterone, *p* = 0.096; figure 3).

**Table 1.** The LMM for the corticosterone release rate regarding the corticosterone type (baseline or stress-induced) before the transfer, habitat of origin, water temperature, interaction between the corticosterone type and the habitat of origin, ID and location. Significant effects are presented in italics.

dependent variable	fixed effect			
corticosterone release rate (pg cm^−1^ h^−1^)		estimate	std error	*p* value
	*stress-induced corticosterone*	*0.467*	*0.134*	*0.001* ***
	*habitat stream*	−*1.159*	*0.211*	0.01*
	interaction between stress-induced corticosterone and habitat stream	0.312	0.189	0.096

### 3.2. After the transfer

**Figure 4.**
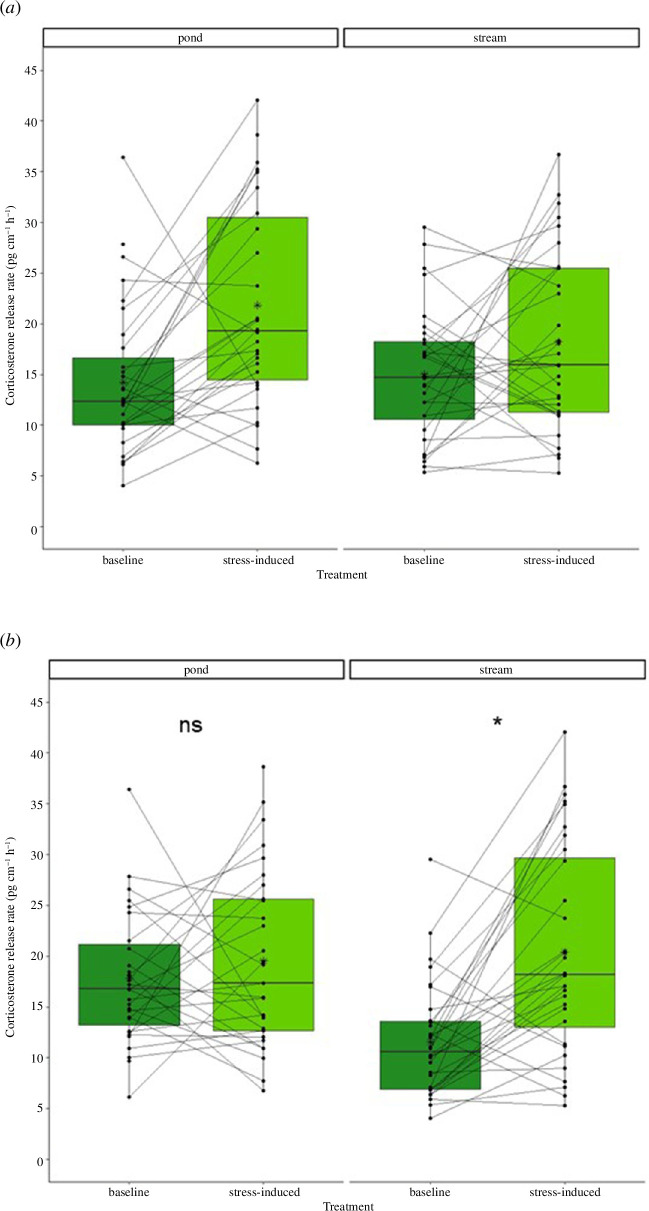
The water-borne corticosterone release rate (pg cm^−1^ h^−1^) in fire salamander larvae (*Salamandra salamandra*) after the transfer experiment. (*a*) According to their habitat of origin (pond: baseline *n* = 32 and stress-induced *n* = 32; stream: baseline *n* = 32 and stress-induced *n* = 31) or (*b*) according to their transfer habitat (pond: baseline *n* = 32 and stress-induced *n* = 31; stream: baseline *n* = 32 and stress-induced *n* = 31). (*a*) Larvae originating from ponds, but not from streams showed a significant increase in stress-induced corticosterone after the transfer (*p* = 0.041). (*b*) Larvae transferred into streams, but not larvae transferred into ponds showed a significant increase in stress-induced corticosterone (*p* = 0.005). Each point represents a single corticosterone measurement and each line connects the two measurements per individual. The horizontal line represents the median and the asterisks in the box represent the mean.

We found a significant interaction between the treatment (i.e. baseline or stress-induced corticosterone) and the original habitat (table 2, *p* = 0.041). While larvae originating from streams did not show an increase in stress-induced corticosterone after the transfer period, larvae originating from ponds did (figure 4). Furthermore, we found an interaction between the treatment (i.e. baseline or stress-induced corticosterone) and the transfer habitat (table 2, *p* = 0.005), with larvae transferred into streams showing a significant increase in stress-induced corticosterone, while larvae transferred into ponds did not show this increase regardless of the habitat of origin (figure 4).

**Table 2.** LMM for the corticosterone release rate regarding the corticosterone type (baseline or stress-induced) after the transfer, habitat of origin, transfer habitat, the interaction between the corticosterone type and the transfer habitat, corticosterone type and habitat of origin and ID and location. Significant effects are presented in italics.

dependent variable	fixed effect			
corticosterone release rate (pg cm^−1^ h^−1^)		estimate	std error	*p* value
	stress-ind. corticosterone	0.474	0.231	0.045*
	transfer habitat (stream)	−0.901	0.484	0.185
	original habitat (stream)	0.074	0.210	0.724
	*interaction between stress-ind. corticosterone and transfer habitat (stream)*	*0.777*	*0.267*	0.005**
	*interaction between stress-ind. corticosterone and original habitat (stream)*	*−0.558*	*0.267*	0.041*

For the transfer habitat pond, we found no difference between baseline and stress-induced corticosterone (*p* = 0.390), while we found a difference for the transfer habitat stream (*p* < 0.05). When comparing the corticosterone types with the transfer habitats, we found no difference between baseline corticosterone (*p* = 0.219) and stress-induced corticosterone (*p* = 0.832).

**Figure 5.**
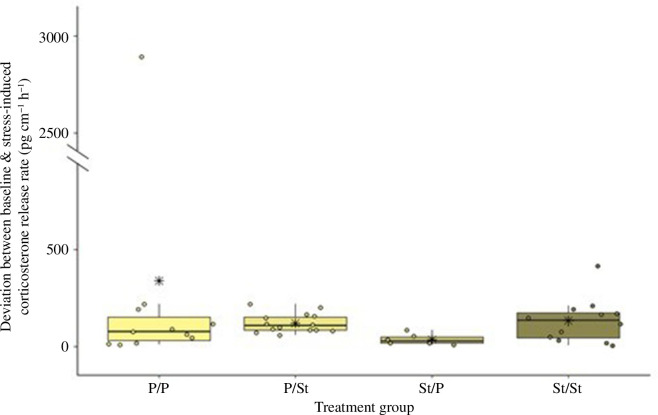
Deviation in baseline and stress-induced corticosterone release rates (pg cm^−1^ h^−1^) between fire salamander larvae from ponds and streams after the transfer for the four treatment groups P/P (*n* = 15), P/St (*n* = 16), St/P (*n* = 15) and St/St (*n* = 16). We found significant differences between P/St and St/P (*p* = 0.007) and between St/P and St/St (*p* = 0.021). Each point represents the deviation in the corticosterone concentration of one individual. The horizontal line represents the median and the asterisks in the box represent the mean.

We found no difference in the deviation between baseline and stress-induced corticosterone in larvae that remained in their original habitat type (St/St and P/P, *p* = 0.92, figure 5). However, larvae that changed their habitat type differed significantly from each other (P/St and St/P, *p* = 0.007). Larvae originating from streams and transferred into ponds (St/P) differed significantly from those that remained in streams (St/St, *p* = 0.021), while we found no difference between larvae that were transferred into streams (P/St) and larvae that remained in streams (St/St, *p* = 0.974). We found no difference between larvae that were transferred from streams to ponds (St/P) and larvae that remained in ponds (P/P, *p* = 0.096).

**Figure 6.**
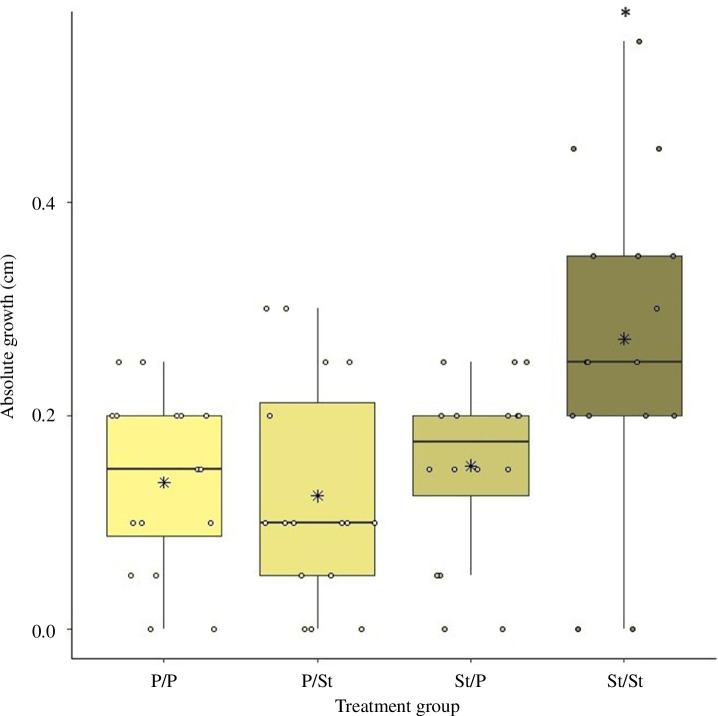
Absolute larval growth (cm) during the transfer period for each treatment group (P/P, P/St, St/P and St/St, each group *n* = 16). St/St differed significantly from all other groups (*p* = 0.004). Each point represents a single measurement, while the horizontal line represents the median and the asterisks in the box represent the mean.

For the growth during the transfer period, we found a significant difference between the transferred groups (*p* = 0.004, figure 6). Larvae that originated from streams and remained in streams (St/St) were the only group that significantly differed from all other groups (St/St and P/P: *p* = 0.019, St/St and P/St: *p* = 0.006 and St/St and St/P: *p* = 0.037, figure 6). We found no difference in larval growth between larvae transferred into ponds (St/P) or streams (P/St) and larvae that remained in ponds (P/P) (St/P and P/St: *p* = 0.917, P/St and P/P: *p* = 0.98 and P/P and St/P: *p* = 0.994).

## 4. Discussion

Our study investigated corticosterone release rates in fire salamander larvae in the field by water-borne hormone sampling. We validated corticosterone measurements for fire salamander larvae as reported by Gabor *et al*. [32]. Moreover, we focused on the stress that the larvae experience in different habitat types and whether they can conform to changing habitat conditions. Environmental changes, as they come with an increase in heavy rainfalls [5] and a higher potential for drought [57], can have different selection pressures on different habitats and fire salamander females usually depositing their larvae into ponds might in the future use streams or vice versa. Thus, understanding whether the larvae can conform to the other habitat is important in terms of conservation.

We showed that pond larvae have significantly higher baseline and stress-induced corticosterone release rates than stream larvae, confirming our first hypothesis. Ponds are not the typical habitat that fire salamander females choose for larval deposition [34]. In ponds, the water is usually warmer and has less oxygen and food, higher predator abundance and a higher rate of conspecifics than in streams [36,39,51]. However, we included water temperature as a fixed effect in our model before the transfer but found no effect between the habitat types. In addition, fire salamander larvae can be cannibalistic, especially when they encounter high densities of conspecifics [58], which is likely to be another stressor for (smaller) larvae. All these factors make ponds the more stressful habitat for fire salamander larvae, which is reflected in their higher corticosterone release rate.

After the salamander transfer, we found an effect of the original habitat and an even stronger effect of the transfer habitat. The larvae transferred into ponds did not show increases in corticosterone release compared with the baseline value, whereas larvae transferred into streams showed an increase in stress-induced corticosterone release. These results indicate that the larvae transferred into streams can react to acute stress, as they showed an increase in the corticosterone release rate after exposure to an acute stressor. We can speculate that this indicates high potential to conform to the habitat stream. In contrast, the larvae transferred into ponds showed no difference between baseline and stress-induced corticosterone release rates any more, indicating the signs of chronic stress and therefore no sign of conformance to the new habitat. Chronic stress leads to the downregulation of the stress-induced corticosterone response towards acute stressors [11,48,59,60], as seen in the larvae that were transferred into ponds. Chronic stress can have severe fitness consequences [61] and a recent study showed that larvae in streams have higher apparent survival than larvae in ponds [62]. Living in a remarkably stressful habitat might be one explanation for this. However, stress may be perceived differently in individuals. Within the treatment groups, we observed strong individual differences in the corticosterone measurements between baseline and stress-induced corticosterone. This might reflect individual differences and therefore distinct individualized niches. From our study site, the larvae differ in their genotype according to the habitat, with most larvae from ponds belonging to the pond genotype and most larvae from streams belonging to the stream genotype [35,46]. However, we did not analyse the genotype of the larvae and thus cannot rule out that the genotype affects individual corticosterone release rates.

Chronic stress can lead to a decreased size during metamorphosis and influence morphology in amphibians, which might influence their survival after metamorphosis. We found that all larvae in ponds and streams were substantially larger after the transfer than before. However, for all larvae that experienced the stressful habitat, the pond, as their habitat of origin, transfer or both, grew less than those that only experienced streams. Increased corticosterone can negatively affect growth, as found in *Rana temporaria* tadpoles (Ruthsatz et al., 2023) and *Hylarana indica* tadpoles [63]. In contrast to our findings, a previous RTE [42] found a higher growth rate in pond larvae. However, they kept the larvae in the transfer treatment for a longer time (mean of 47 days versus 14 days in this study). This suggests that growth might be initially faster in stream larvae but with time faster in pond larvae. Changing abiotic factors over time might also play a role, as Oswald and Caspers [42] started their transfer experiment in March, while we conducted our study at the end of April. Considering larval physiology is especially important for conservation purposes and the larval stage can be considered the most critical life stage during fire salamander ontogeny. Larval habitats are already heavily affected by changing climate conditions such as higher temperatures leading to desiccation. Thus, as larvae from both habitat types can conform when transferred into streams, streams should be considered for conservation actions in the future to enhance larval physiological health. Future research should focus on potential physiological differences between pond and stream larvae, e.g. the influence on the larval immune system, and the impact that these habitats might have on larval fitness. In addition, this newly validated method can be used to test the influence of other factors on larval physiology such as pollution, temperature and food availability.

## 5. Conclusion

Fire salamander larvae from ponds and streams differ in several aspects such as their morphology and behaviour [50,64,65]. However, less is known about potential differences in physiological health between larvae from different habitats. We demonstrated that larvae in ponds have a higher baseline and stress-induced corticosterone release rate than those in streams. However, larvae transferred into ponds showed a downregulated stress response indicating an acute stressor, while those transferred into streams did not. These results indicated that (i) ponds are a more stressful environment for fire salamander larvae, (ii) corticosterone release rates are flexible across environments, and (iii) the larvae can, if at all, only partially conform to a given habitat. In addition, with this study, we validated the use of water-borne corticosterone for fire salamander larvae and thus enabled its future use from different perspectives that might influence larval physiology.

## Data Availability

Data and code can be found here [66].
